# Small-Molecule Inhibitor Leads of Ribosome-Inactivating Proteins Developed Using the Doorstop Approach

**DOI:** 10.1371/journal.pone.0017883

**Published:** 2011-03-24

**Authors:** Yuan-Ping Pang, Jewn Giew Park, Shaohua Wang, Anuradha Vummenthala, Rajesh K. Mishra, John E. McLaughlin, Rong Di, Jennifer Nielsen Kahn, Nilgun E. Tumer, Laszlo Janosi, Jon Davis, Charles B. Millard

**Affiliations:** 1 Computer-Aided Molecular Design Laboratory, Mayo Clinic, Rochester, Minnesota, United States of America; 2 Department of Plant Biology and Pathology, School of Environmental and Biological Sciences, Rutgers University, New Brunswick, New Jersey, United States of America; 3 Division of Biochemistry, Walter Reed Army Institute of Research, Silver Spring, Maryland, United States of America; Auburn University, United States of America

## Abstract

Ribosome-inactivating proteins (RIPs) are toxic because they bind to 28S rRNA and depurinate a specific adenine residue from the α-sarcin/ricin loop (SRL), thereby inhibiting protein synthesis. Shiga-like toxins (Stx1 and Stx2), produced by *Escherichia coli*, are RIPs that cause outbreaks of foodborne diseases with significant morbidity and mortality. Ricin, produced by the castor bean plant, is another RIP lethal to mammals. Currently, no US Food and Drug Administration-approved vaccines nor therapeutics exist to protect against ricin, Shiga-like toxins, or other RIPs. Development of effective small-molecule RIP inhibitors as therapeutics is challenging because strong electrostatic interactions at the RIP•SRL interface make drug-like molecules ineffective in competing with the rRNA for binding to RIPs. Herein, we report small molecules that show up to 20% cell protection against ricin or Stx2 at a drug concentration of 300 nM. These molecules were discovered using the doorstop approach, a new approach to protein•polynucleotide inhibitors that identifies small molecules as doorstops to prevent an active-site residue of an RIP (*e.g.*, Tyr80 of ricin or Tyr77 of Stx2) from adopting an active conformation thereby blocking the function of the protein rather than contenders in the competition for binding to the RIP. This work offers promising leads for developing RIP therapeutics. The results suggest that the doorstop approach might also be applicable in the development of other protein•polynucleotide inhibitors as antiviral agents such as inhibitors of the Z-DNA binding proteins in poxviruses. This work also calls for careful chemical and biological characterization of drug leads obtained from chemical screens to avoid the identification of irrelevant chemical structures and to avoid the interference caused by direct interactions between the chemicals being screened and the luciferase reporter used in screening assays.

## Introduction

Shiga toxin (Stx) produced by the bacteria *Shigella dysenteriae* and Shiga-like toxins (Stx1 and Stx2) produced by certain strains of *Escherichia coli* are potent ribosome-inactivating proteins (RIPs) [1]. Shiga-like-toxin–producing *E. coli* O157:H7 is an emerging bacterial pathogen responsible for outbreaks of foodborne disease with significant morbidity and mortality in the United States [2]. *E. coli* O157:H7 is the most common cause of hemolytic uremic syndrome, causing more than 20,000 infections and as many as 250 deaths annually [3]. Ricin is another potent RIP isolated from the seeds of the widely available castor plant, *Ricinus communis* [4], belonging to a family of dichain cytotoxins (type II RIPs) that includes abrin and several other plant toxins [5]. While not frequently associated with disease, the toxicity of ricin has made it an attractive tool for both bioterrorism and the targeted killing of cancerous cells [4].

Type II RIPs have two subunits: subunit A, which binds to 28S ribosomal RNA (rRNA) and depurinates a specific adenine residue from the α-sarcin/ricin loop (SRL) thereby inhibiting protein synthesis [6], [7], and subunit B, which recognizes specific receptors on the target cell and facilitates transfer of subunit A into the cell where the inhibition of ribosome activity occurs [8]. According to site-directed mutagenesis and X-ray diffraction studies along with a transition-state analysis of the depurination caused by ricin subunit A (RTA) [9]–[13], the catalytic mechanism of depurination by RTA begins with sandwiching of the adenine ring of the substrate rRNA between Tyr80 and Tyr123 of RTA via pi-pi interactions [9] at the Michaelis-Menten state [14]. These interactions enable the protonation of the adenine ring at N3 [9] by the cationic Arg180 of RTA that forms a hydrogen bond to the anionic Glu177 of RTA at the transition state [10]. The protonation consequently cleaves the adenine in the zwitterionic form from the ribose by breaking the bond between N9 of the adenine and C1 of the ribose, thus leading to the formation of a cationic ribose intermediate stabilized by Glu177 at the transition state. A water molecule activated by the neutral Arg180 subsequently attacks the ribocation to form the ribose product and resume the cationic Arg180 [10]–[13].

**Figure 1:**
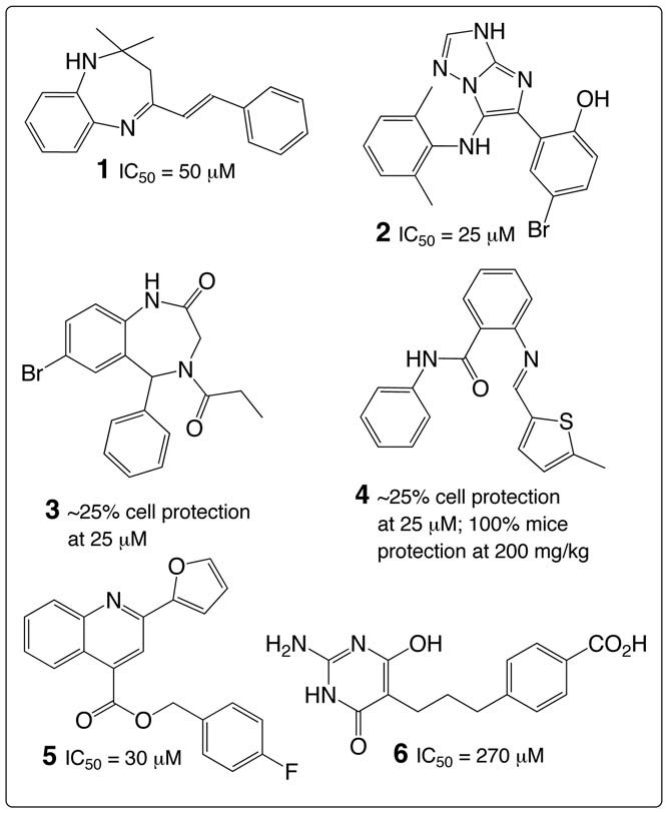
Known small-molecule inhibitors of ribosome-inactivating proteins.

Small-molecule inhibitors of ricin and Shiga/Shiga-like toxins are sought for potential pre-exposure or post-exposure treatment of RIP poisoning. Additionally, because ricin and abrin have potential medical use as immunotoxin components [15], small-molecule inhibitors can also serve as co-treatments to control immunotoxin toxicity. Some oligonucleotides developed through structure-based design—circular DNA and DNA/RNA hybrid molecules, for example—inhibit RTA at nanomolar concentrations [16]–[19]; the most potent has a *K*_i_ value of 2.3 nM [19] and is potentially useful in the immunotoxin cancer therapy [15], [19]. These molecules are effective in neutralizing extracellular toxin to prevent further intoxication, but they generally cannot enter cells to neutralize intracellular toxin. Small-molecule inhibitors of RTA and Shiga/Shiga-like toxins identified from high-throughput screens (HTSs) inhibit toxin transport at various stages at micromolar concentrations [20], [21]; at a concentration of 200 mg/kg, one such inhibitor demonstrated full protection of mice against a dose of ricin that kills 90% of the unprotected control mouse population (**1**–**4** in Figure 1) [21]. Because of the lack of structural and regulatory information about components involved in toxin transport, optimization of these transport inhibitors can be difficult.

Both HTS and structure-based approaches have been pursued in the search for small molecules that can penetrate cells to neutralize intracellular toxins by inhibiting subunit A1 of Stx2 (Stx2A1) or RTA [22]–[25]. The brute force approach has culminated in a small-molecule inhibitor of RTA with a half maximal inhibitory concentration (IC_50_) value of 30 µM (**5** in Figure 1) [22]; the rational approach has led to an inhibitor with an IC_50_ value of 270 µM (**6** in Figure 1) [25]. Clearly, formidable challenges lie in the path of the structure-based design of molecules that can enter cells to inhibit RIPs directly. In our view, one key challenge is due to strong electrostatic interactions at the RIP•SRL interface [26]–[29] that make drug-like molecules ineffective competitors with polynucleotides for binding to RIPs because drug-like molecules are not highly charged.

In this article, we report the discovery of promising small molecules that demonstrate in vitro and ex vivo inhibition of Stx2 and ricin, using a novel approach to small-molecule inhibitors of protein•polynucleotide functions. This approach circumvents the challenge of the strong electrostatic interactions at the RIP•SRL interface. We discuss insights derived from these leads into structure-based design of improved RIP inhibitors, potential application of the new approach to other protein•polynucleotide-function inhibitors, and caveats for using chemical screens to uncover drug leads.

## Results

### RIP Inhibitors Identified Using a Virtual Screen

**Figure 2:**
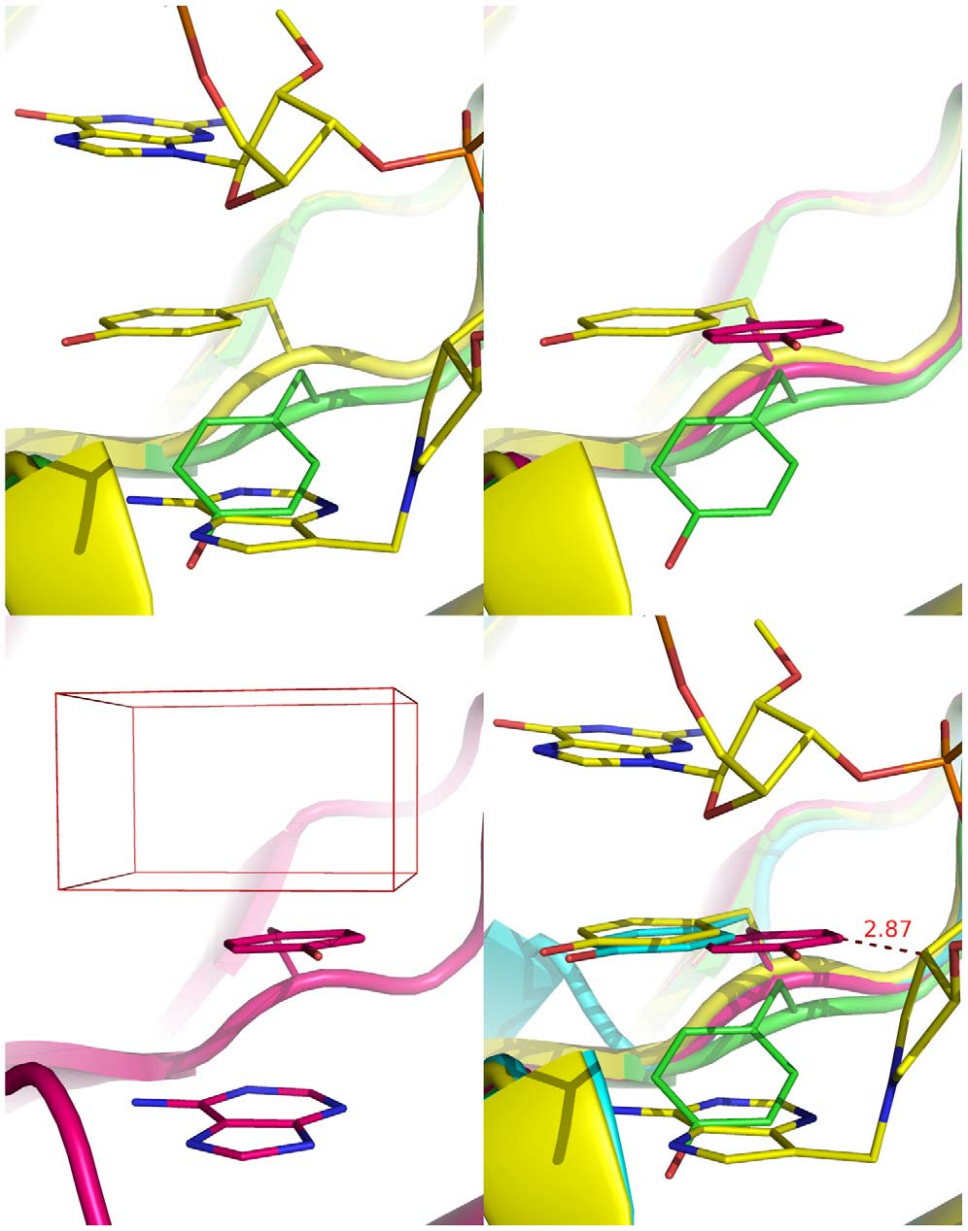
Tyr80 in crystal structures of ricin subunit A (RTA) in the bound and unbound states. a (top left): overlay of the *apo* RTA (green, 1IFT [32]) with the oligonucleotide-bound RTA at the Michaelis-Menten state (yellow; 3HIO [13]) showing that the adenine group markedly perturbs the conformation of Tyr80; b (top right): three distinct conformations of Tyr80: conformations 1, 2, and 3 represent the *apo* conformation in green (1IFT [32]), the less populated bound conformation in magenta (1IFS [32]), and the most populated bound conformation in yellow (1FMP [31]), respectively; c (bottom left): the phenolic ring with an adenine group underneath and a docking box atop in the less populated bound conformation (1IFS [32]); d (bottom right): overlay of the oligonucleotide-bound RTA at the Michaelis-Menten state (yellow; 3HIO [13]) with RTA in conformation 1 (green; 1IFT [32]), conformation 2 (magenta; 1IFS [32]), and conformation 3 (cyan; 1FMP [31]) showing the closeness of the Tyr80 conformations in 3HIO and 1FMP and the clash between the nucleotide and Tyr80 in 1IFS.

Site-directed mutagenesis and kinetic studies as well as the X-ray crystallographic analysis [24], [30]–[32] show that, upon binding to the RTA active site, the adenine group of the SRL substrate markedly changes the side-chain conformation of Tyr80, a catalytically important active-site residue of RTA. As a result of this conformational change, the phenolic ring of Tyr80 can stack with the adenine group and catalysis proceeds (Figure 2a), whereas the side-chain conformation of another catalytically crucial active-site residue of RTA, Tyr123, remains unchanged upon the rRNA binding.

Informed by these seminal findings and the aforementioned challenge of obtaining protein•polynucleotide-interaction inhibitors, we decided to use a doorstop approach to identify small-molecule inhibitors of RTA and Stx2. This new approach aims to identify small molecules that work as doorstops to prevent an active-site residue of an RIP (*e.g.*, Tyr80 of ricin or Tyr77 of Stx2) from adopting the active conformation thereby blocking the function of the protein rather than work as contenders in the competition for binding to the RIP.

We analyzed 13 RTA crystal structures that were available at the time of our virtual screen (described below) and identified three distinct side-chain conformations of Tyr80 (Figure 2b, conformations 1–3). Conformation 1 represents the Tyr80 conformation in *apo*-RTA crystal structures (Protein Data Bank [PDB] IDs: 1IFT [32], 1RTC [33], 1IL5 [24], and 2AAI [34]). Conformation 2 is a less populated conformation of Tyr80 in the bound state, found in inhibitor-bound RTA crystal structures (PDB IDs: 1IFS [32] and 1APG [31]). Conformation 3 shows the most populated Tyr80 conformation in the bound state, found in crystal structures of RTA in complex with various adenine analogs (PDB IDs: 1BR5 [23], 1BR6 [23], 1IL3 [24], 1IL4 [24], 1IL9 [24], 1IFU [32], and 1FMP [31]). We conjectured that conformation 3 is the active conformation necessary for catalysis and that molecules capable of preventing Tyr80 from adopting this conformation could inhibit RTA without direct competition with SRL for binding to the RTA active site.

In this context, we performed the following virtual screen to identify small molecules that can bind at the active site of the RTA crystal structure of 1IFS [32] to stabilize conformation 2 thereby preventing its conversion to conformation 3. Our reason to stabilize conformation 2 instead of conformation 1 was that an adenine molecule can fit underneath the phenolic ring of Tyr80 in conformation 2 (Figure 2c) and permits the use of a clip-like molecule to stabilize conformation 2 with two functional groups simultaneously binding on both sides of the phenolic ring, whereas conformation 1 lacks space for an adenine-like molecule beneath the phenolic ring.

Using an automated computer docking program (EUDOC) [35]–[37], we screened 236,925 small molecules for molecules that bind favorably in the region enclosed by a docking box over the phenolic ring of Tyr80 in the 1IFS crystal structure (Figure 2c) [32]. This screen identified 226 chemicals with EUDOC energies (intermolecular interaction energies) lower than −50 kcal/mol. All of the small molecules screened were selected from an in-house database of 2.5 million chemicals using the criterion that molecular weight must not be greater than 300 Da. Typically, we select chemicals using two energy criteria: (1) the EUDOC energy must be <−40 kcal/mol, and (2) the van der Waals component of the EUDOC energy must be <−25 kcal/mol. These criteria are derived from our observation that all experimentally confirmed micromolar inhibitors identified by EUDOC have EUDOC energies and the van der Waals components below these values [38]–[40]. Because the protein•polynucleotide interaction in this study is mainly electrostatic [26]–[29], we lowered the EUDOC energy cutoff to −50 kcal/mol and removed the van der Waals component criterion.

**Figure 3:**
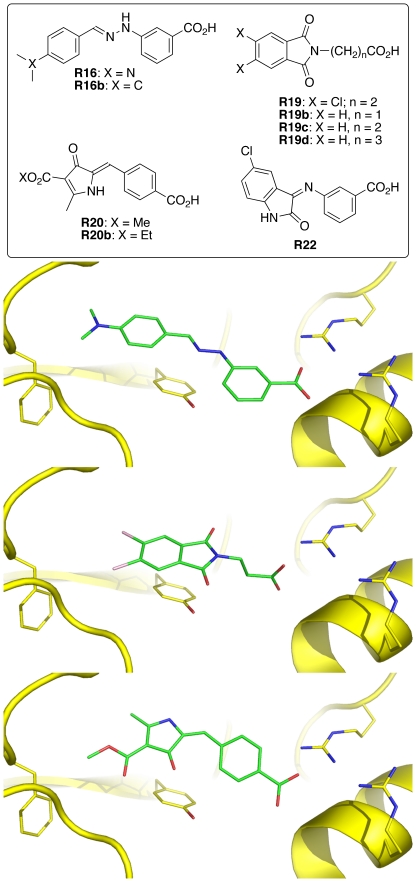
Chemical structures and binding modes of small-molecule inhibitors of ricin subunit A. The complexes were generated using the EUDOC program with the 1IFS crystal structure [32]. The residues from left to right are Phe93, Tyr80, Arg258, and Arg213.

We visually inspected the 226 selected molecules using the following criteria to identify those with the characteristics required to stabilize conformation 2: (1) a carboxylate group that mimics the phosphate group of the rRNA substrate to interact with Arg213 and Arg258 of RTA, (2) an aromatic ring that has the off-center pi-pi interaction with Tyr80, and optionally (3) an alkyl group that forms the van der Waals interaction with the methylene group of Phe93 (Figure 3). We then weeded out those that were commercially unavailable or have multiple chiral centers, poor solubility, or poor cell permeability. Subsequently, we purchased 27 compounds for biological evaluation.

### Syntheses of RIP Inhibitors

After an initial biological evaluation of the 27 purchased chemicals, we synthesized the four most promising inhibitors, **R16**, **R19**, **R20**, and **R22** (Figure 3) for spectroscopic analyses that require relatively large quantities of materials or for making close analogues (**R16b**, **R19b–d**, and **R20b**; Figure 3). Although **R16**, **R19**, and **R20** were commercially available from SPECS (www.specs.net), and **R22** was available from ASINEX (www.asinex.com), synthesis or spectroscopic data for **R16**, **R16b**, **R20**, **R20b**, and **R22** have not hitherto been reported.

**Figure 4:**
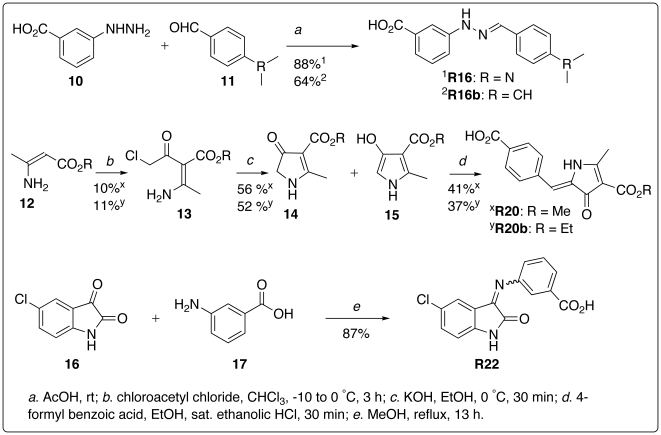
Synthetic schemes for R16, R20, and R22.

**R16** and **R16b** were made in good yields by reacting 3-hydrazinobenzoic acid in acetic acid with benzaldehydes (Figure 4). **R19** and its analogues were readily prepared according to a known procedure [41]. **R22** was obtained from a reaction of 5-chloroindoline-2,3-dione with 3-aminobenzoic acid in methanol (Figure 4). The proton NMR spectra of **R16**, **R19**, and **R22** prepared in house are identical to those of the chemicals purchased from SPECS and ASINEX. The proton NMR and NOESY experiments show that **R22** exists in a mixture of *E* and *Z* stereoisomers with the Z isomer being dominant, which is consistent with the *Z* stereochemistry of **R22** used in our virtual screen.

**Figure 5:**
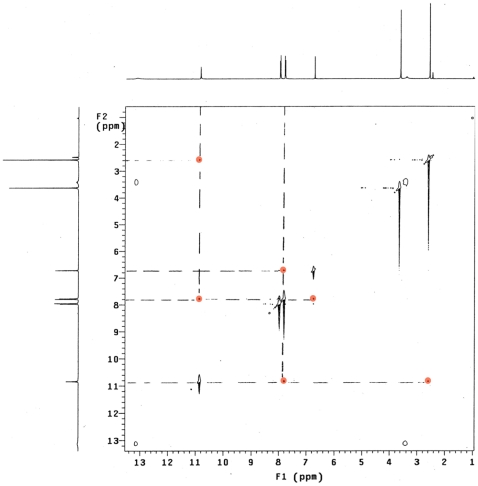
NOESY spectrum of R20 indicating the keto form and the *Z* stereochemistry.

**R20** or **R20b** was prepared by coupling 4-formylbenzoic acid with a substituted pyrrole in the keto form for **R20** or a mixture of keto and enol forms for **R20b** according to a reported process [42] (Figure 4). The substituted pyrrole was obtained via cyclization of 2-amino-2-(2-chloroacetyl)butenoate [43], which was prepared from 3-aminobutenoate using a literature procedure [44]. **R20** has the *E* stereochemistry according to the chemical structure specified by SPECS (catalog number AO-081/14455020). The proton NMR spectrum of **R20** made in house matches that of **R20** purchased from SPECS. Furthermore, the in vitro and ex vivo biological activities of the in-house and purchased **R20** were the same. However, the NOESY spectrum shows that the in-house **R20** exists in the *Z* stereochemistry because of our observed correlations of the nitrogen-attached proton with the methyl and phenyl protons in **R20** (Figure 5). Consistent with the *Z* stereochemistry of **R20**, (*Z*)-ethyl 2-methyl-5-(4-nitrobenzylidene)-4-oxo-4,5-dihydro-1*H*-pyrrole-3-carboxylate—a close analog of **R20b**—has been reported to have the *Z* stereochemistry [45]. Therefore, identification of **R20** as an active RIP inhibitor resulted from sheer luck, because the *E* stereochemistry of **R20** specified by the chemical vendor was used in our virtual screen.

### Evaluation of RIP Inhibitors Using in Vitro and ex Vivo Methods

**Figure 6:**
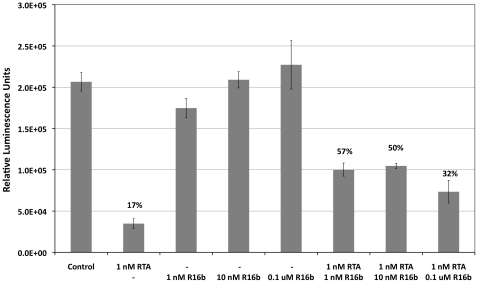
Concentration effects of R16b on the luciferase activity.

**Figure 7:**
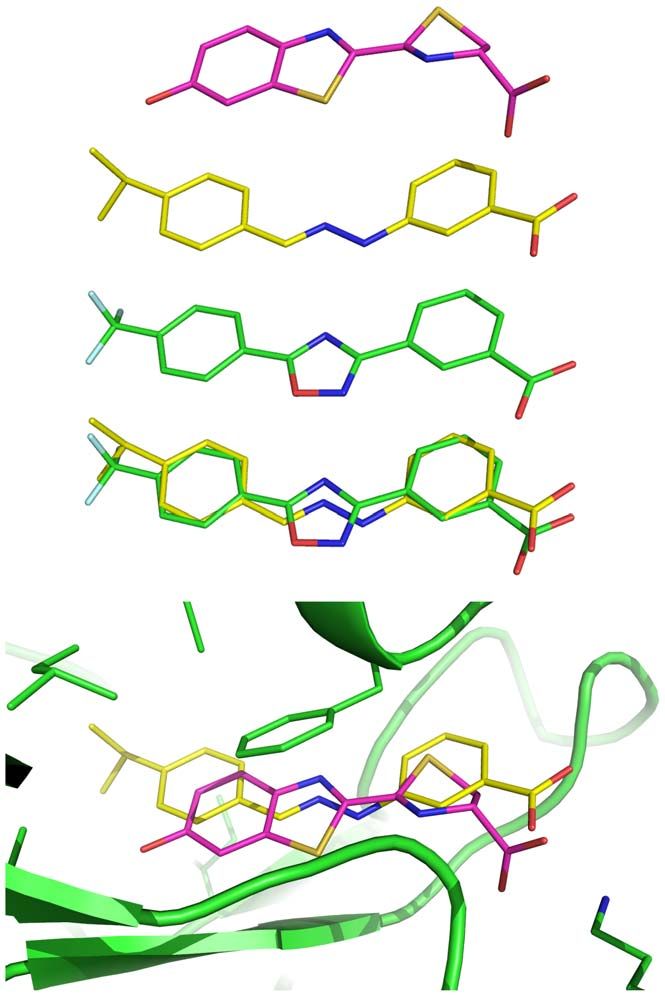
Structural similarity of R16b to D-luciferin and 3-(5-(4-(trifluoromethyl)phenyl)-1,2,4-oxadiazol-3-yl)benzoic acid. Row 1: D-luciferin; Row 2: **R16b**; Row 3: 3-(5-(4-(trifluoromethyl)phenyl)-1,2,4-oxadiazol-3-yl)benzoic acid; Row 4: overlay of **R16b** and 3-(5-(4-(trifluoromethyl)phenyl)-1,2,4-oxadiazol-3-yl)benzoic acid; Row 5: **R16b** or D-luciferin bound in the active site of firefly luciferase. Conformations of **R16b**, D-luciferin, and 3-(5-(4-(trifluoromethyl)phenyl)-1,2,4-oxadiazol-3-yl)benzoic acid in the free state were energy optimized by *ab initio* calculations at the HF/6-31G*//HF/6-31G* level using the Gaussian 98 program [62].

Firefly-luciferase–based cell-free translation assays with rabbit reticulocyte lysate (RRL) [46] confirmed that 22 of the 27 compounds identified in our virtual screen showed some degrees of RTA inhibition at an inhibitor concentration of 50 nM. Of the 22 active compounds, **R16**, **R19**, **R20**, and **R22** were the most promising. Further studies of these inhibitors and their analogs (**R16b**, **R19b**, **R19c**, **R19d**, and **R20b**) showed a 1.1- to 1.7-fold increase in luciferase activity resulting from the translation in the RRL after treatment with 1 nM RTA and 1 nM inhibitor, relative to the activity after treatment with 1 nM RTA only (Table 1). **R19b** and **R16b** showed 1.7- and 1.6-fold increases in luciferase activity, respectively. Interestingly, the luciferase activity in the RRL treated with **R16b** alone increased as the concentration of **R16b** increased, whereas that of the RRL treated with RTA and **R16b** decreased as the **R16b** concentration increased (Figure 6). Other inhibitors showed similar concentration effects on luciferase activity. These concentration effects made the determination of IC_50_ values difficult and suggested that these inhibitors might interact with both RTA and firefly luciferase owing to the structural similarity of the inhibitors such as **R16b** to D-luciferin that is the substrate of firefly luciferase [47] and to 3-(5-(4-(trifluoromethyl)phenyl)-1,2,4-oxadiazol-3-yl)benzoic acid that is a known inhibitor of firefly luciferase [48] (Figure 7).

**Table 1:** Increase of rabbit reticulocyte lysate in vitro translation caused by exposure to 1 nM ribosome-inactivating protein inhibitor and 1 nM ricin subunit A (RTA) or 0.5 nM Shiga-like toxin 2 (Stx2) relative to the exposure to 1 nM RTA or 0.5 nM Stx2.

Inhibitor	Translation (%)	Translation (%)	Fold	Translation (%)	Translation (%)	Fold
	RTA	RTA+Inhibitor	Improvement	Stx2	Stx2+Inhibitor	Improvement
**R16**	32	34	1.1	35	38	1.1
**R16b**	30	48	1.6	35	48	1.4
**R19**	32	36	1.1	35	43	1.2
**R19b**	30	51	1.7	35	46	1.3
**R19c**	30	47	1.6	35	44	1.3
**R19d**	30	48	1.6	35	38	1.1
**R20**	32	38	1.2	35	46	1.3
**R20b**	30	41	1.4	35	47	1.3
**R22**	30	42	1.4	35	44	1.3

**Figure 8:**
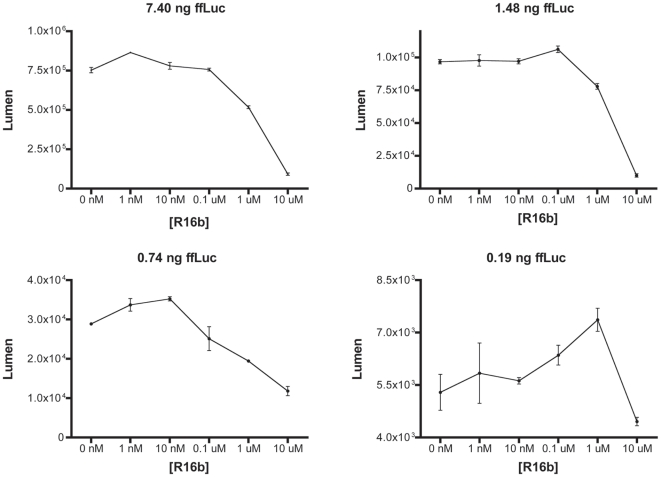
Concentrational effect of R16b on the firefly luciferase (ffLuc) activity. Upper panel: the activity of high-concentration ffLuc versus **R16b** concentration. Lower panel: the activity of low-concentration ffLuc versus **R16b** concentration.

Twenty 10-ns (1.0-fs time step) molecular dynamics simulations of the **R16b**-bound firefly luciferase showed that **R16b** binds at the luciferase active site in almost the same way as D-luciferin does (Figure 7 and Datasets S1 and S2). The average intermolecular interaction energy calculated from 20,000 conformations of the **R16b**-bound luciferase obtained from the simulations is of −134.2 kcal/mol, whereas the corresponding energy computed from 7,000 conformations of D-luciferin-bound enzyme obtained from seven 10-ns (1.0-fs time step) molecular dynamics simulations is −102.0 kcal/mol. These results suggested that **R16b** could bind to the luciferase active site. Indeed, subsequent experimental studies showed that **R16b** had two direct effects on the firefly luciferase activity in a dose-dependent manner. As apparent from Figure 8, **R16b** first increased and then decreased the luciferase activity as the concentration of **R16b** gradually changed from 0 to 10 µM, and this bell-shaped dose response is most noticeable when the luciferase concentration is low (0.74–0.19 ng of firefly luciferase). These results demonstrate that **R16b** has direct interactions with firefly luciferase.

To confirm that our compounds directly inhibit RTA as well, we performed cell titer 96 AQuaeous non-radioactive cell proliferation assays (Promega, Madison, Wisconsin) using Sp2 mouse myeloma cells [49] to test the ability of these compounds to protect cells against ricin. This assay determines cell viability by measuring the absorbance of the formazan product produced by viable cells rather than detecting cellular adenosine 5′-triphosphate (ATP) levels through the use of firefly luciferase. At a concentration of 300 nM, these inhibitors showed 0.7–15.7% cell protection against ricin (Table 2), confirming that these compounds are capable of inhibiting RTA in the absence of firefly luciferase. Most of these inhibitors showed similar cell protections at 3 µM, 30 µM, and 300 nM (Table 2), suggesting possible interactions of these inhibitors at high concentrations with other off-targets in the Sp2 mouse myeloma cells.

**Table 2:** Ex vivo inhibition of ribosome-inactivating proteins by R16, R19, R20, and R22 and their analogues at 0.3, 3.0, and 30.0 µM.

Inhibitor	% cell protection (SD) by 0.3 µM inhibitor against ricin	% cell protection (SD) by 3.0 µM inhibitor against ricin	% cell protection (SD) by 30.0 µM inhibitor against ricin	% cell protection by 0.3 µM inhibitor against Stx2
**R16**	13 (6)	22 (5)	21 (7)	17
**R16b**	12 (6)	16 (1)	22 (2)	11
**R19**	13 (7)	15 (4)	16 (3)	16
**R19b**	16 (5)	19 (4)	15 (2)	11
**R19c**	15 (5)	20 (2)	18 (2)	8
**R19d**	9 (5)	13 (4)	10 (5)	3
**R20**	5 (6)	4 (3)	8 (3)	15
**R20b**	2 (9)	4 (9)	8 (6)	6
**R22**	1 (6)	0 (4)	1 (6)	21

**Figure 9:**
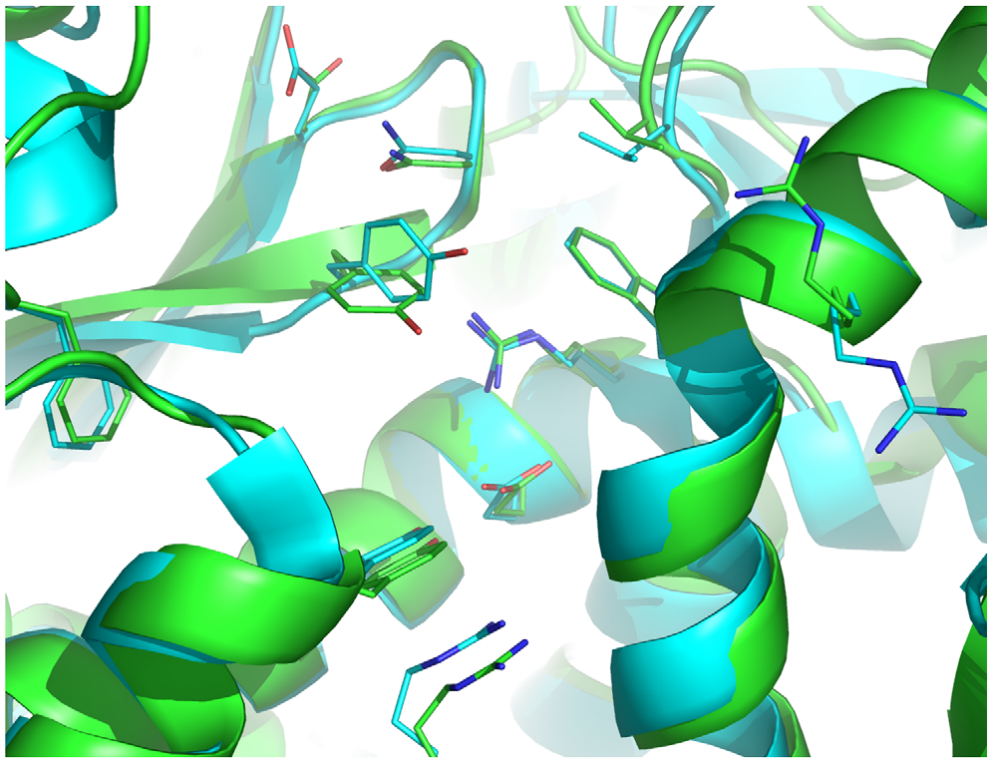
Overlay of ricin subunit A (RTA) and Shiga-like toxin 2 subunit A1 (Stx2A1). The RTA (green) and Stx2A1 (yellow) structures are taken from crystal structures of 1IFS [32] and 1R4P [50].

Superimposition of the crystal structures of RTA (PDB ID: 1IFS [32]) and Stx2 (PDB ID: 1R4P [50]) showed that the conformations of active-site residues of Stx2A1 are similar to those of RTA (Figure 9), although the sequence identity of the two proteins is only 18%. This similarity prompted us to test our inhibitors against Stx2 both in vitro and ex vivo. Indeed, **R16b** and **R22** demonstrated 1.4- and 1.3-fold increases in luciferase activity resulting from the translation in the RRL after treatment with 0.5 nM Stx2 (activated by reacting with trypsin and dithiothreitol and 1 nM inhibitor) relative to the activity after treatment with 0.5 nM activated Stx2 only (Table 1). Furthermore, **R22** showed 21% cell protection against Stx2 at a drug concentration of 300 nM. Of mechanistic importance, among the nine inhibitors tested, **R22** is the most potent in inhibiting Stx2, whereas **R19b** is the most potent in inhibiting ricin at the same drug concentration, which demonstrates the preferential interactions of the tested inhibitors with RTA and Stx2A1.

## Discussion

### Promising RIP Inhibitor Leads

We have two lines of evidence for the significant and direct inhibition of ricin and Stx2 by our inhibitors at an inhibitor concentration of 1 or 300 nM, despite the unexpected interactions between our inhibitors and firefly luciferase. First, these inhibitors showed two different structure-activity relationships for RTA and activated Stx2 assayed under the same conditions involving firefly luciferase and substrate D-luciferin. If the observed enhancement of the firefly luciferase activity by our inhibitors were solely due to the interaction with the luciferase, it would be highly improbable to have two different structure-activity relationships (Table 1). Second, our inhibitors exhibited up to 20% cell protection against ricin or activated Stx2 under the assay conditions that are devoid of the luciferase.

Inhibitors of firefly luciferase are known to have the ability to increase the luciferase activity through a modest increase of the enzyme half-life by reversible binding to the enzyme [48], [51], [52]. In this study we found that **R16b** first increases and then decreases the low-concentration luciferase activity as the concentration of **R16b** changes from 0 to 10 µM (lower panel of Figure 8). Although further experimental verification is needed, these results may explain why the luciferase activity in the RRL treated with **R16b** alone increases as the concentration of **R16b** increases, whereas the luciferase activity in the RRL treated with RTA and **R16b** decreases as the **R16b** concentration increases (Figure 6). When the RRL was treated with 1–100 nM **R16b** alone, **R16b** was presumably in shortage relative to an ample amount of firefly luciferase resulting from the translation in the RRL. In this case, a relatively small amount of **R16b** could modestly enhance the half-life of the enzyme but would not be enough to inhibit the binding of D-luciferin substrate to the enzyme; therefore, **R16b** could increase the luciferase activity. When the RRL was treated with 1 nM RTA and 1–100 nM **R16b** that inhibits RTA, **R16b** was presumably in excess relative to a residual amount of the luciferase resulting from the translation in the RRL that was incompletely inhibited by RTA in the presence of **R16b** in low concentrations. In this case, a relatively large amount of **R16b** decreased the enzymatic activity because the inhibition of D-luciferin binding to the enzyme by **R16b** outweighs the increase of the luciferase half-life by **R16b**.

Taken together, our cell-free and cell-based studies as well as the hypothetical mechanisms of the inhibitor concentration effects on the luciferase activity suggest that **R16**, **R16b**, **R19**, **R19b**, **R19c**, **R19d**, **R20**, **R20b**, and **R22** are promising inhibitor leads of ricin and Stx2.

### The Doorstop Approach to Inhibiting Protein•Polynucleotide Functions

In this work, we used the doorstop approach to identify small molecules that work as doorstops to prevent the active-site residue Tyr80 in RTA from adopting its active conformation, thereby blocking the function of the protein rather than work as a contender in the binding competition. Further studies are needed to validate the approach and determine the scope of its application. At this stage, informed by the fortuitous finding of **R20** as an RIP inhibitor and the complication of unexpected interactions with firefly luciferase, it is reasonable to question whether the identification of **R16**, **R19**, and **R22** as RIP inhibitor leads resulted from sheer luck or the use of the doorstop approach. For the following reasons, we attribute the finding of these leads to the doorstop approach.

Our screening work hinges on the assumption that conformation 2 is a bound conformation that inhibits catalysis. If this assumption were incorrect, our RIP inhibitor discovery would be serendipitous. As apparent from the superimposition of the crystal structure of RTA liganded with a cyclic tetranucleotide (PDB ID: 3HIO [13]) over the crystal structures of RTAs in complex with small-molecule inhibitors (PDB IDs: 1IFS [32], 1IFT [32], and 1FMP [31]) shown in Figure 2d, Tyr80 in conformation 2 (found in the 1IFS crystal structure) clashes with the nucleotide of the 3HIO crystal structure at the Michaelis-Menten state, whereas Tyr80 in conformation 3 (found in the 1FMP crystal structure) overlays well with that of the 3HIO crystal structure. The clash of Tyr80 in conformation 2 with the nucleotide substrate supports the assumption that conformation 2 inhibits catalysis. This suggests that the discovery of the RIP inhibitors was likely conferred by the doorstop approach and that the doorstop approach might be useful for developing inhibitors of other protein•polynucleotide functions.

### Potential Applications of the Doorstop Approach

Without knowing whether a molecule can grip conformation 2 by binding atop the phenolic ring of Tyr80, we initially wanted to design a clip-like molecule to stabilize conformation 2 using two functional groups simultaneously binding on both sides of the phenolic ring. For this reason, we performed virtual screening using the 1IFS crystal structure with conformation 2 [32], which has space to accommodate an adenine-like molecule underneath the phenolic ring of Tyr80, rather than using the 1IFT crystal structure with conformation 1 [32], which lacks space beneath the ring. However, conformation 2 may not be readily available for other protein**•**polynucleotide complexes. Its rarity may raise a concern about the generality of the doorstop approach. Interestingly, our in vitro and ex vivo testing results show that molecules such as **R16b**, **R19b**, and **R22** with functional groups binding on one side of the phenolic ring appear to be effective doorstops, suggesting that effective doorstops might be obtainable from screening chemicals for their binding atop the Tyr80 ring in conformation 1, which is a common *apo*-state conformation. In this context, it is worth noting the following potential application of the doorstop approach.

The *N*-terminal domain of the E3L protein of vaccinia virus binds Z-DNA and causes pathogenicity in mice [53]. In addition, the side-chain conformation of Tyr48 at the Z-DNA binding site of E3L is in an equilibrium of UP (c = x+0°) and DOWN (c = x+180°) states; the E3L protein with Tyr48 in the UP state cannot bind Z-DNA and does not cause pathogenicity whereas the one in the DOWN state binds Z-DNA and causes pathogenicity [54]. Furthermore, the amino acid sequence of vaccinia virus E3L is almost identical to those of poxviruses [55]. These results suggest that an active-site Tyr residue may serve as a common switch for functions of protein•polynucleotide complexes. The doorstop approach might be applicable for the development of other protein•polynucleotide inhibitors—such as small-molecule inhibitors of Z-DNA binding proteins in poxviruses—as antiviral agents.

### Caveats for the Use of Chemical Screens for Potential Drug Leads

In our previous virtual screen for farnesyltransferase inhibitors, we found that six out of 27 compounds purchased from chemical vendors had serious chemical identity or purity issues [38]. In this study, of the two chemicals (**R20** and **R22**) that required spectroscopic analyses to confirm their stereochemistry, one (**R20**) turned out to have incorrect stereochemistry. These results call for careful chemical characterization of leads identified from chemical screens using spectroscopic analyses to avoid issues of chemical identity and purity.

Having long been used in HTSs for drug leads, luciferase is a reporter that detects the transcriptional activity in translation assays and measures cellular ATP levels in cell viability assays or kinase activity assays [56]. However, only recently has attention been directed to interference caused by direct interactions between luciferase and chemicals being screened [48], [51], [52], [57]. The experimentally confirmed direct interaction of **R16b** with firefly luciferase reported herein reinforces the reported advocation that appropriate control studies must be performed before interpreting HTS results [48].

### Conclusion

Using the doorstop approach that aims to identify molecules that can prevent an RIP active-site residue from adopting an active conformation, we identified chemicals with significant in vitro inhibitory potency at nanomolar concentrations and up to 20% cell protection against ricin and Stx2 at an inhibitor concentration of 300 nM. This work offers promising leads for structural optimization to achieve better cell protection. The results suggest that the doorstop approach might be applicable to the development of small-molecule inhibitors of poxvirus Z-DNA binding proteins as anti-poxvirus agents. This work also calls for careful chemical and biological characterization of leads obtained from HTSs to avoid identification of irrelevant chemical structures and interference caused by unintended interactions with luciferase.

## Materials and Methods

### Reagents and Toxins

Hexanes (Hex), ethyl acetate (EtOAc), and trifluoroacetic acid (TFA) were purchased from Fisher Scientific (Pittsburgh, PA). All commercially available reagents such as dimethyl sulfoxide (DMSO) were used as received. Rabbit reticulocyte cell-free lysate (2 parts water and 1 part lysate) was obtained from Green Hectares (Oregon, WI). Ricin and RTA were purchased from Vector Laboratories (Burlingame, CA). Stx2 holotoxin was provided by the Phoenix Laboratory (Tufts-NEMC Microbial Products & Services Facility). Hybridoma Serum Free Medium, Glutamax, and a mixture of penicillin and streptomycin were obtained from Invitrogen (Carlsbad, CA). The Cell Titer 96 AQuaeous Non-Radioactive Cell Proliferation Assay reagents were purchased from Promega (Madison, WI).

### Chemical Synthesis

#### General Description

The ^1^H NMR (400 MHz) and ^13^C NMR (100 MHz) spectra were recorded on a Mercury 400 spectrometer from Varian (Palo Alto, CA). Chemical shifts are reported in ppm using either tetramethylsilane or the solvent peak as an internal standard. Data are reported as follows: chemical shift, multiplicity (s = singlet, brs = broad singlet, d = doublet, t = triplet, brt = broad triplet, q = quartet, h = septet, m = multiplet), coupling constant, and integration. Low-resolution mass spectra were recorded using either Hewlet Packard 5973 Mass Spectrometer with SIS Direct Insertion Probe (Palo Alto, CA) or Waters ZQ/EMD 1000 Mass Spectrometer (Milford, MA). High-resolution mass spectra were obtained on a Bruker BioTOF II ESI. IR spectra were obtained on a ThermoNicolet Avatar 370 FT-IR (Waltham, MA) using KBr pellet. Medium pressure liquid chromatography (MPLC) was performed with Biotage SP-1 (Charlottesville, VA) using silica gel (EM Science, 230–400 mesh). The salt form of the compounds used for biological testing was prepared quantitatively by treating the acid in methanol with one equivalent of 0.5 M NaHCO_3_ solution.

#### (*E*)-3-(2-(4-(Dimethylamino)benzylidene)hydrazinyl)benzoic acid (R16)

To a stirred suspension of 3-hydrazinobenzoic acid (0.50 g, 3.29 mmol) in acetic acid (10 mL) was added 4-(dimethylamino)benzaldehyde (0.49 g, 3.29 mmol) at room temperature. After stirring for 16 hours at the same temperature, the bright yellow precipitate was collected by filtration, the filter cake was washed with acetic acid and then with water, and dried under high vacuum to afford 0.82 g (88%) of the titled compound as a yellow powder. The purity of **R16** determined by HPLC (Zorbax SB C-18, 250×4.6 mm, 1.0 mL/min, *t*_R_ = 12.25 minutes, gradient at 80% A to 100% B over 20 minutes, solvent A = H_2_O with 0.1% TFA, solvent B = 1∶9/H_2_O∶MeCN with 0.1% TFA) was 97.63% (see Figure S1). mp 239–245°C (decomp); ^1^H NMR (DMSO-*d*_6_) δ 12.76 (brs, 1H), 10.14 (s, 1H), 7.77 (s, 1H), 7.57 (s, 1H), 7.46 (d, *J* = 8.6 Hz, 2H), 7.29–7.19 (m, 3H), 6.72 (d, *J* = 8.6 Hz, 2H), and 2.92 (s, 6H); ^13^C NMR (DMSO-*d*_6_) δ 168.38, 151.10, 146.67, 139.34, 132.21, 129.90, 127.66, 124.00, 119.43, 116.41, 112.95, 112.77, and 40.60; IR cm^−1^ 3306, 2967, 1678, 1609, 1511, and 1303; LRMS-EI *m*/*z* 283 (100%, [M^+^]), 147 (28%, [M−C_9_H_11_N_2_^+^]); Anal. calcd for C_16_H_17_N_3_O_2_: C, 67.83; H, 6.05; N, 14.83. Found: C, 67.67; H, 6.06; N, 14.76.

#### (*E*)-3-(2-(4-Isopropylbenzylidene)hydrazinyl)benzoic acid (R16b)

To a stirred suspension of 3-hydrazinobenzoic acid (0.50 g, 3.29 mmol) in acetic acid (10 mL) was added 4-isopropylbenzaldehyde (90% purity, 0.55 mL, 3.29 mmol) at room temperature. After stirring 50 minutes at the same temperature, the precipitate was collected by filtration, the filter cake was washed with ice-cooled acetic acid and then with ice-cooled 95% EtOH, and dried under high vacuum to afford 0.59 g (64%) of the titled compound as a white powder, which became pink in color in 1 hour at room temperature. The purity of the compound determined by HPLC (Zorbax SB C-18, 250×4.6 mm, 1.0 mL/min, *t*_R_ = 21.10 minutes, gradient at 80% A to 100% B over 20 minutes, solvent A = H_2_O with 0.1% TFA, solvent B = 1∶9/H_2_O∶MeCN with 0.1% TFA) was 99.04% (see Figure S2). mp 208–211°C; ^1^H NMR (DMSO-*d*_6_) δ 12.81 (brs, 1H), 10.44 (s, 1H), 7.85 (s, 1H), 7.62 (s, 1H), 7.56 (d, *J* = 8.0 Hz, 2H), 7.31–7.24 (m, 5H), 2.87 (h, *J* = 6.8 Hz, 1H), and 1.18 (d, *J* = 6.8 Hz, 6H); ^13^C NMR (DMSO-*d*_6_) δ 168.27, 149.32, 146.25, 138.23, 133.92, 132.30, 130.00, 127.33, 126.51, 120.10, 116.70, 113.18, 33.98, and 24.46; IR (KBr) cm^−1^ 3299, 3007, 2954, 1683, 1585, 1487, 1291, and 1123; LRMS-EI *m*/*z* 282 (100%, [M^+^]), 267 (30%, [M−CH_3_^+^]); Anal. calcd for C_17_H_18_N_2_O_2_: C, 72.32; H, 6.43; N, 9.92. Found: C, 72.28; H, 6.35; N, 9.91.

#### 3-(5,6-Dichloro-1,3-dioxoisoindolin-2-yl)propanoic acid (R19) [58]

To a stirred solution of 4,5-dichlorophthalic anhydride (1.08 g, 5.0 mmol) in acetic acid (5 mL) was added β-alanine (0.44 g, 5.0 mmol) and refluxed for 16 hours. The reaction mixture was cooled to room temperature, and the precipitate was filtered, washed with water, and dried under high vacuum to give **R19** as a white solid (1.20 g, 84%). mp 244–246°C; ^1^H NMR (DMSO-*d*_6_) δ 12.40 (brs, 1H), 8.17–8.15 (m, 2H), 3.76 (t, *J* = 7.6 Hz, 2H), and 2.58 (t, *J* = 7.6 Hz, 2H); ^13^C NMR (DMSO-*d*_6_) δ 172.72, 166.51, 137.94, 132.28, 125.91, 34.61, and 32.79. IR cm^−1^ 3485, 3101, 1785, 1720, 1438, 1389, 1225, and 1119; LRMS-EI *m*/*z* 287 (15%, [M^+^]), 270 (16%), 241 (100%), and 228 (82%); HRMS-ESI 309.9662 ([M+Na^+^], C_11_H_7_Cl_2_NO_4_Na^+^ requires 309.9650). Anal. calcd for C_11_H_7_Cl_2_NO_4_: C, 45.86; H, 2.45; N, 4.86. Found: C, 46.01; H, 2.73; N, 4.89.

#### 2-(1,3-Dioxoisoindolin-2-yl)acetic acid (R19b) [59]

Prepared using the procedure for making **R19**. Anal. calcd for C_10_H_7_NO_4_: C, 58.54; H, 3.44; N, 6.83. Found: C, 58.64; H, 3.58; N, 6.77.

#### 3-(1,3-Dioxoisoindolin-2-yl)propanoic acid (R19c) [41]

Prepared using the procedure for making **R19**. Anal. calcd for C_11_H_9_NO_4_: C, 60.27; H, 4.14; N, 6.39. Found: C, 60.22; H, 4.14; N, 6.32.

#### 4-(1,3-Dioxoisoindolin-2-yl)butanoic acid (R19d) [41]

Prepared using the procedure for making **R19**. Anal. calcd for C_12_H_11_NO_4_: C, 61.80; H, 4.75; N, 6.01. Found: C, 61.89; H, 4.57; N, 5.95.

#### (*E*)-Methyl 3-amino-2-(2-chloroacetyl)but-2-enoate (13x)

To a cooled solution of methyl crotonate **12x** (20 g, 173.7 mmol) in chloroform (250 mL) at −10°C was added dropwise chloroacetyl chloride (34.5 mL, 434.2 mmol) and the resulting solution was then stirred at 0°C for 3 hours. The reaction mixture was neutralized with sodium carbonate, washed with water, dried over anhydrous MgSO_4_, filtered and the solvent was removed in *vacuo*. The crude product was washed with hot hexanes (3×200 mL) to afford the desired product **13x** (3.3 g, 10%) as a white crystalline solid. mp 232–238°C (decomp); ^1^H NMR (DMSO-*d*_6_) δ 10.56 (brs, 1H), 8.79 (s, 1H), 4.46 (s, 2H), 3.62 (s, 3H), and 2.17 (s, 3H); ^13^C NMR (DMSO-*d*_6_) δ 189.63, 170.92, 169.07, 99.31, 51.56, 49.90, and 23.13; IR cm^−1^ 3318, 3183, 2950, 1695, 1597, 1450, 1303, 1209, 1127, 1029, 812, 767, and 710; LRMS-EI *m*/*z* 191 ([M^+^], 10%), and 142 ([M−CH_2_Cl^+^], 100%). HRMS-ESI 192.0422 ([M+H^+^], C_7_H_11_ClNO_3_ requires 192.0427).

#### Methyl 2-methyl-4-oxo-4,5-dihydro-1*H*-pyrrole-3-carboxylate (14x)

To a vigorously stirred solution of KOH (220 mg, 3.92 mmol) in anhydrous EtOH (2 mL) was added compound **13x** (300 mg, 1.57 mmol) in one portion. The reaction mixture was immediately cooled to 0°C. The suspension was then stirred at room temperature for 30 minutes. The reaction mixture was then acidified with ice cold 1 N HCl and the solid thus obtained was filtered and washed with water to give the desired compound **14x** as a white solid (135 mg, 56%). mp 208–212°C (decomp); ^1^H NMR (DMSO-*d*_6_) δ 9.37 (s, 1H), 3.80 (s, 2H), 3.55 (s, 3H), and 2.38 (s, 3H); ^13^C NMR (DMSO-*d*_6_) δ 193.67, 181.85, 164.66, 101.17, 55.09, 50.41 and 17.30; IR cm^−1^ 3146, 2946, 1712, 1621, 1561, 1507, 1454, 1368, 1193, 1078, and 1054; LRMS-EI *m/z* 155 ([M^+^], 67%); HRMS-ESI 178.0458 ([M+Na^+^], C_7_H_9_NO_3_Na^+^ requires 178.0480).

#### (*Z*)-4-((4-(Methoxycarbonyl)-5-methyl-3-oxo-1*H*-pyrrol-2(3*H*)-ylidene)methyl)benzoic acid (R20)

A suspension of **14x** (200 mg, 1.29 mmol) in EtOH (25 mL) was slowly added to a solution 4-formylbenzoic acid (102 mg, 1.29 mmol) in EtOH (50 mL containing 1 mL of conc. HCl). The solution turned yellow rapidly. The reaction mixture was cooled to 0°C, the yellow precipitate was filtered and washed with ethanol to yield the desired compound **R20** (152 mg, 41%) as a yellow solid. The Beilstein and silver nitrate test for halogen showed that the reaction product **R20** did not contain chloride, thus confirming that **R20** exists as an anion at pH of 7.4. mp 285–290°C (decomp); ^1^H NMR (DMSO-*d*_6_) δ 13.14 (brs, 1H), 10.85 (s, 1H), 7.97 (d, *J* = 8.4 Hz, 2H), 7.79 (d, *J* = 8.4 Hz, 2H), 6.73 (s, 1H), 3.63 (s, 3H), and 2.58 (s, 3H); ^13^C NMR (DMSO-*d*_6_) δ 181.94, 176.76, 167.49, 164.03, 137.80, 134.29, 131.48, 131.01, 130.31, 113.71, 102.89, 50.94, and 16.68; IR cm^−1^ 3354, 2950, 1704, 1614, 1565, 1479, 1389, 1225, 1193, 1115, 1078, 788 and 700; LRMS-EI *m/z* 287 ([M^+^], 45%), and 228 ([M−C_2_H_3_O_2_^+^], 100%); HRMS-ESI 288.0864 ([M+H^+^], C_15_H_14_NO_5_ requires 288.0872). Anal. calcd for C_15_H_13_NO_5_: C, 62.72; H, 4.56; N, 4.88. Found: C, 62.40; H, 4.94; N, 4.80.

#### (*E*)-Ethyl 3-amino-2-(2-chloroacetyl)but-2-enoate (13y) [43]

Prepared using the procedure for making **13x**. Compound **13y** (3.5 g, 11%) was obtained as a white crystalline solid from ethyl crotonate **12y** (20 g, 154.85 mmol) and chloroacetyl chloride (31 mL, 387.10 mmol). mp 132–134°C (decomp); ^1^H NMR (DMSO-*d*_6_) δ 10.56 (brs, 1H), 8.78 (s, 1H), 4.46 (s, 2H), 4.10 (q, *J* = 7.2 Hz), 2.17 (s, 3H), and 1.21 (t, *J* = 7.2 Hz); ^13^C NMR (DMSO-*d_6_*) δ 189.61, 170.87, 168.62, 99.57, 60.26, 49.80, 23.16, and 14.69; IR cm^−1^ 3318, 3191, 2987, 1691, 1626, 1458, 1287, 1205, 1148, 1042, 764 and 706; LRMS-EI *m/z* 205 ([M^+^], 10%), 156 ([M−CH_2_Cl^+^], 100%), and 128 ([M−C_2_H_2_ClO^+^], 50%); HRMS-ESI 228.0388 ([M+Na^+^], C_8_H_12_ClNO_3_Na^+^ requires 228.0403).

#### Ethyl 2-methyl-4-oxo-4,5-dihydro-1*H*-pyrrole-3-carboxylate (14y) and ethyl 4-hydroxy-2-methyl-1*H*-pyrrole-3-carboxylate (15y) [60]

Prepared using the procedure for making **14x**. In this case the product was extracted with CH_2_Cl_2_ after acidification with dilute HCl. Intermediates **14y** and **15y** (213 mg, 52%) were synthesized from **13y** (500 mg, 2.43 mmol) and KOH (341 mg, 6.08 mmol) as a tautomeric mixture (2∶3). mp 160–170°C (decomp); ^1^H NMR (DMSO-*d_6_*) δ 10.67 (s, 1H), 9.33 (s, 1H), 7.64 (s, 2H), 6.04 (d, *J* = 2.0 Hz, 1H), 4.16 (q, *J* = 7.2 Hz, 2H), 4.04 (q, *J* = 7.2 Hz, 3H), 3.79 (s, 3H), 2.38 (s, 4H), 2.28 (s, 3H), 1.23 (t, *J* = 7.2 Hz, 3H), and 1.16 (t, *J* = 7.2 Hz, 4H); ^13^C NMR (DMSO-*d*_6_) δ 193.80, 181.71, 166.75, 164.12, 145.66, 131.43, 101.37, 100.35, 98.68, 98.67, 59.40, 58.58, 55.05, 17.82, 15.18, 15.09, and 14.22; IR cm^−1^ 2357, 1699, 1470, 1328, 1291, 1234, 1180, 1074, 1029, 849 and 805; LRMS-EI *m*/*z* 169 ([M^+^], 44%); HRMS-ESI 170.0803 ([M+H^+^], C_8_H_12_NO_3_ requires 170.0817).

#### (*Z*)-4-((4-(Ethoxycarbonyl)-5-methyl-3-oxo-1*H*-pyrrol-2(3*H*)-ylidene)methyl)benzoic acid (R20b)

**R20b** (132 mg, 37%) was synthesized from a mixture of **14y** and **15y** (200 mg, 1.18 mmol) and 4-formyl benzoic acid (178 mg, 1.18 mmol) according to the procedure of making **R20** and the anionic state of **R20b** at pH of 7.4 was confirmed by the Beilstein and silver nitrate test. mp 195–198°C (decomp); ^1^H NMR (DMSO-*d*_6_) δ 13.13 (brs, 1H), 10.81 (s, 1H), 7.97 (d, *J* = 8.0 Hz, 2H), 7.79 (d, *J* = 8.4 Hz, 2H), 6.70 (s, 1H), 4.11 (q, *J* = 7.2 Hz, 2H), 2.58 (s, 3H) and 1.21 (t, *J* = 6.8 Hz, 3H); ^13^C NMR (DMSO-*d*_6_) δ 182.09, 176.53, 167.49, 163.47, 137.81, 134.30, 131.44, 130.99, 130.31, 113.50, 103.09, 59.25, 16.77 and 15.09; IR cm^−1^ 3415, 2970, 1708, 1601, 1556, 1499, 1384, 1189, 1115, 1078, 1004, 800 and 670. LRMS-EI *m*/*z* 301 ([M^+^], 65%); HRMS-ESI 324.0842 ([M+Na^+^], C_16_H_15_NO_5_Na^+^ requires 324.0848). Anal. calcd for C_16_H_15_NO_5_•1.2 H_2_O: C, 59.51; H, 5.43; N, 4.34. Found: C, 59.30; H, 5.19; N, 4.47.

#### 3-(5-Chloro-2-oxoindolin-3-ylideneamino)benzoic acid (R22)

5-Chloroindoline-2,3-dione (546 mg, 3 mmol) and 3-aminobenzoic acid (411 mg, 3 mmol) were added into 20 mL dry MeOH. The mixture was stirred at reflux for 13 hours and filtered through a filter paper. The solid was washed with MeOH to give pure 3-(5-chloro-2-oxoindolin-3-ylideneamino)benzoic acid (781 mg, 87%) as an orange solid, which contained *E* and *Z* isomers. mp 326–327°C (decomp); ^1^H NMR (DMSO-*d*_6_) δ 13.10 (brs, 1H), 11.12 (s, 1.02H), 11.00 (s, 0.42H), 7.84 (d, *J* = 7.6 Hz, 1.02H), 7.68 (d, *J* = 7.6 Hz, 0.43H), 7.63–7.38 (m, 4.80H), 7.27–7.23 (m, 1.46H), 6.90 (d, *J* = 8.4 Hz, 1.02H), 6.87 (d, *J* = 8.4 Hz, 0.43H), and 6.14 (s, 1H); ^13^C NMR (DMSO-*d*_6_) δ 167.85, 167.48, 163.77, 158.96, 155.47, 153.64, 150.88, 149.59, 146.62, 145.27, 134.62, 134.41, 132.91, 131.74, 130.85, 129.38, 127.16, 126.74, 126.19, 125.92, 125.32, 124.17, 123.53, 123.20, 122.61, 120.42, 118.65, 117.53, 113.92, and 113.15; IR cm^−1^ 3195, 1716, 1683, 1622, 1442, 1307, and 1209; LRMS-EI *m/z* (%): 300 ([M^+^], 50%), 272 ([M−H_2_O^+^], 100%); Anal. calcd for C_15_H_9_ClN_2_O_3_: C, 59.91; H, 3.02; N, 9.32. Found: C, 59.77; H, 2.88; N, 9.27.

### *in Silico* Screening

The two-stage docking of 236,925 small molecules into the active site of RTA was carried out by using the EUDOC program [35]–[37] according to a published protocol [35]. The translational and rotational increments at the first stage were 1.0 Å and 10 degrees of arc, respectively, and default increments were used at the second stage. A cutoff of −50 kcal/mol for intermolecular interaction energies was used. The 236,925 small molecules were selected from an in-house database of 2.5 million small molecules using the criterion that each selected molecule has a molecular weight less than 301. All small molecules to be screened were protonated or deprotonated according to physiological pH of 7.4 and their three-dimensional structures and atomic charges were obtained from AM1 semi-empirical calculations. Conformations of RTA and small molecules were not allowed to change during the docking. A docking box (6.0×3.5×6.0 Å^3^) was defined to confine the translation of the mass centre of each molecule within the active site of RTA crystal structure (PDB ID: 1IFS [32]). The box was surrounded by Asp100, Ile-104, Asp75, Asn78, Tyr80, Val82, Phe93, Gly120, Gly121, Asn122, His94, Pro95, and Asp96 whose conformations were defined in the 1IFS structure (see Figure 2c). All water molecules and the bound adenine were removed from the 1IFS structure. The docking studies were performed on a dedicated cluster of 470 Xeon Processors (2.2/2.4 GHz).

### Simulations of Firefly Luciferase Liganded with R16b or D-luciferin

#### Model Preparation

The atomic charges of **R16b** and D-luciferin were generated according to the RESP procedure [61] with *ab initio* calculations at the HF/6–31G*//HF/6–31G* level using the Gaussian 98 program [62]. The force field parameters including the charges of **R16b** and D-luciferin are included in Datasets S1 and S2, respectively. The starting structure of firefly luciferase in complex with **R16b** was generated by manually docking **R16b** into the active site of the luciferase structure that was taken from the crystal structure of luciferase bound with 5′-*O*-[*N*-(dehydroluciferyl)-sulfamoyl]adenosine (PDB ID: 2D1S [47]). In the manual docking, the carbonyl carbon atom of **R16b** was placed near the carbonyl carbon atom of D-luciferin in the 2D1S crystal structure, and the alkyl-substituted aromatic carbon atom was placed near the hydroxy-substituted aromatic carbon of the D-luciferin structure. The starting structure of luciferase in complex with D-luciferin was extracted from the 2D1S crystal structure. For the luciferase structure, all histidine residues were treated as HID, and crystallographically determined water molecules were removed. The topology and coordinate files of luciferase in complex with **R16b** or D-luciferin were generated by the PREP, LINK, EDIT, and PARM modules of the AMBER 5.0 program [63]. The complex was refined by energy minimization using a dielectric constant of 1.0 and 100 cycles of steepest-descent minimization followed by 100 cycles of conjugate-gradient minimization. The refined complex was solvated with 13,617 and 13,540 TIP3P water molecules (named WAT) [64] for **R16b** and D-luciferin, leading to a system of 12,802 and 12,842 atoms, respectively. The WAT molecules were obtained from solvating the complex using a pre-equilibrated box of 216,000 TIP3P molecules, whose hydrogen atom charge was set to 0.4170, where any water molecule was removed if it had an oxygen atom closer than 2.2 Å to any solute atom or a hydrogen atom closer than 2.0 Å to any solute atom, or if it was located further than 8.2 Å along the x-, y-, or z-axis from any solute atom.

#### Multiple Molecular Dynamics Simulations

The solvated complex system was energy-minimized for 100 cycles of steepest-descent minimization followed by 100 cycles of conjugate-gradient minimization to remove close van der Waals contacts in the system, then heated from 0 to 300 K at a rate of 10 K/ps under constant temperature and volume, and finally simulated independently with a unique seed number for initial velocities at 300 K under constant temperature and pressure using the PMEMD module of the AMBER 8.0 program [65] with the AMBER force field (ff99SB) [66], [67]. All simulations used (1) a dielectric constant of 1.0, (2) the Berendsen coupling algorithm [68], (3) a periodic boundary condition at a constant temperature of 300 K and a constant pressure of 1 atm with isotropic molecule-based scaling, (4) the Particle Mesh Ewald method to calculate long-range electrostatic interactions [69], (5) a time step of 1.0 fs, (6) the SHAKE-bond-length constraints applied to all the bonds involving the H atom, (7) saving the image closest to the middle of the “primary box” to the restart and trajectory files, (8) formatted restart file, and (9) default values of all other inputs of the PMEMD module. Twenty different molecular dynamics simulations (each lasted 10 ns) were carried out for luciferase in complex with **R16b**, and seven difference simulations were performed for luciferase bound with D-luciferase.

#### Structure Analysis

The average structure of the simulations for each luciferase complex was obtained by using the PTRAJ module of the AMBER 11 program [65]. The average intermolecular interaction energy for each complex was obtained using an in-house program (ISE, wrote by Yuan-Ping Pang) that computes the average of the intermolecular interaction energies of all trajectories saved at the 1-ps intervals during the last 1-ns period of the 20 (for the **R16b** complex) or 7 (for the D-luciferin complex) different simulations.

### Cell-Free Assay for Inhibition of RTA and Stx2

#### *N*-Glycosidase Preparation

A stock solution of RTA (7.5 µM) was prepared using phosphate buffered saline solution (PBS). A stock solution of activated Stx2 was prepared according to published reports [70]–[72] with slight modifications. Briefly, lyophilized Stx2 powder was re-suspended in PBS to prepare a 2-µM solution. From this solution, 2 µg of Stx2 was incubated with trypsin (prepared in 1 mM HCl, pH 3.0 and used at a final concentration of 2 ng/µL) and 30 mM dithiothreitol for 2 hours at 37°C. The treatment with trypsin was stopped by adding phenylmethylsulfonyl fluoride at a final concentration of 0.04 µg/µL. The concentrated stock solutions of RTA and activated Stx2 were maintained at 4°C. The diluted working solutions of the two *N*-glycosidases were disposed after each experiment.

#### Inhibitor Preparation

**R16**, **R19**, and **R20** in their acid form were dissolved in neat DMSO to a final concentration of 10 mM. Stock solutions of these compounds (1 mM) were prepared in 10% DMSO. The final DMSO concentration for the *in vitro* translation reaction assay was set at 0.67% DMSO. **R16b**, **R19b**, **R19c**, **R19d**, **R20b**, and **R22** in their sodium salt form were dissolved in double distilled water at 5.06 mM, 60 mM, 1.41 M, 37 mM, 0.31 M, and 46 mM, respectively. Stock solutions of the water-soluble compounds were prepared at 1 mM.

#### Cell-Free Translation Assay

The final protein concentration of the diluted RRL was adjusted to approximately 80 mg/mL as measured using a spectrophotometer (OD_280_). The lysate was treated with micrococcal nuclease according to a published procedure [46]. An RRL master mix was prepared by supplementing with the same buffer and ATP regeneration system used for yeast *in vitro* translation assays [46]. Uncapped luciferase RNA was produced using the Epicenter AmpliScribe T7 kit (AS3107). A total of 1 µg uncapped luciferase RNA per 30 µL reaction and added to the RRL master mix just prior to running the assay. The test inhibitors were pre-incubated with RTA or activated Stx2 for 30 minutes at room temperature prior to being added to the wells containing the RRL master mix. RTA and activated Stx2 for the toxin only treatments were preincubated with either DMSO for the DMSO compound comparisons or PBS buffer for the water soluble compound comparisons. Reactions, setup in 96-well polymerase-chain-reaction plates, were incubated at 30°C for 1 hour. Following the one-hour incubation, the reaction was stopped by adding 100 µL Tris buffered saline. Using multichannel pipets, 10 µL of the diluted reactions were added to white 96-microwell plates (Nunc 236105) for the luminometer assay. The amount of active luciferase protein (indicating translation efficiency of the *in vitro* reaction) was measured using the Biotek 96-well-plate luminometer. The system was programmed such that the automatic injector added 100 µL of Promega's Luciferase Assay Reagent (E1501) to each well and read with a 2 second delay, 10 second integrated light measurement. Samples were run in triplicate in at least two independent experiments. The data was analyzed using Microsoft Excel.

### Cell-Based Assay for Protection against Ricin

Mouse myeloma Sp2/0-Ag14 cells (CRL-1581, American Type Culture Collection, Manassas, Virginia) were pre-grown to early-mid log phase in Hybridoma Serum Free Medium (HSFM) supplemented with 4 mM Glutamax and 0.5% (v/v) penicillin and streptomycin mix. Cells were collected with low-speed centrifugation (1,500 rpm in a Sorvall RT-6000 centrifuge, Thermo Electron Corp., Ashville, NC) at 4°C for 15 minutes, resuspended in fresh HSFM and plated in the wells of 96-well sterile microplates (Corning Costar 3595, Corning Incorporated, Corning, NY) to result in 2.5e+5/mL final cell density. The cells were then incubated in the absence of any other additives (Viability Control), in the presence of 40 pg/mL ricin (Ricin Inhibition Control), in the presence of 0.3, 3 and 30 µM RIP inhibitor test substance (Substance Toxicity Control) and in the combined presence of the above amounts of ricin and inhibitor test substances (Test) in 5% CO_2_ atmosphere with 100% relative humidity at 37°C for 16 hours. A mixture of MTS and PMS reagents from the Cell Titer 96 AQuaeous Non-Radioactive Cell Proliferation Assay was added to the cells according to the manufacturer's recommendations and the plates were read at 490 nm after further incubation for 4 hours. The data were transformed by subtracting the OD_490_ data obtained with the Ricin Inhibition Control from all OD_490_ values where ricin was present. Cell viabilities in the Test wells were calculated by expressing the OD_490_ values in percent of the OD_490_ values of Viability Control wells (% Viability). We show the mean values from 8 parallel experiments along with the standard deviation of the data in parentheses.

### Cell-Based Assay for Protection against Stx2

The assay was similar to Sp2 cell proliferation assay to test ricin inhibitors for ricin antagonism with the exception that Vero cells (ATCC CCL-81) (green African monkey) replaced Sp2/0-Ag14 [73]. Vero cells were maintained in EMEM medium (Eagle's minimum essential medium) supplemented with 10% (FBS) fetal bovine serum, 20 unit/ml penicillin and 20 mg/mL streptomycin mix (Invitrogen, Carlsbad, California) and cultured at 37°C with 5% CO_2_. The cells were incubated in the absence of any other additives (Viability Control), in the presence of 50 ng/mL Stx2 (Stx2 Inhibition Control), in the presence of the test substance (30, 3 and 0.3 µM) (Substance Toxicity Control) and in the combined presence of the above amounts of Stx2 and test substances (Test) in 5% CO_2_ atmosphere with 100% relative humidity at 37°C for 10 hours. MTS/PMS reagent from the Cell Titer 96 AQuaeous Non-Radioactive Cell Proliferation Assay (Promega, Madison, Wisconsin) mix was added to the cells following the manufacturer's recommendation and plates were read spectrophotometrically at 490 nm after a 1–2 hr incubation. The cell protection levels of substances to Stx2 intoxication were calculated similarly as those to ricin.

## Supporting Information

Figure S1 **R16 HPLC chromatogram.**
(PDF)

Figure S2 **R16b HPLC chromatogram.**
(PDF)

Dataset S1 **The energy-minimized average conformation of R16b-bound firefly luciferase complex generated by the multiple molecular dynamics simulations.**
(PDB)

Dataset S2 **The energy-minimized average conformation of the D-luciferin-bound firefly luciferase complex generated by the multiple molecular dynamics simulations.**
(PDB)
