# Supplementary figures and images for: Small-Molecule Inhibitor Leads of Ribosome-Inactivating Proteins Developed Using the Doorstop Approach
Source: PLoS One. 2011 Mar 24;6(3):e17883. doi: 10.1371/journal.pone.0017883 (PMC3063779; doi:10.1371/journal.pone.0017883)

# R16 HPLC Chromatogram

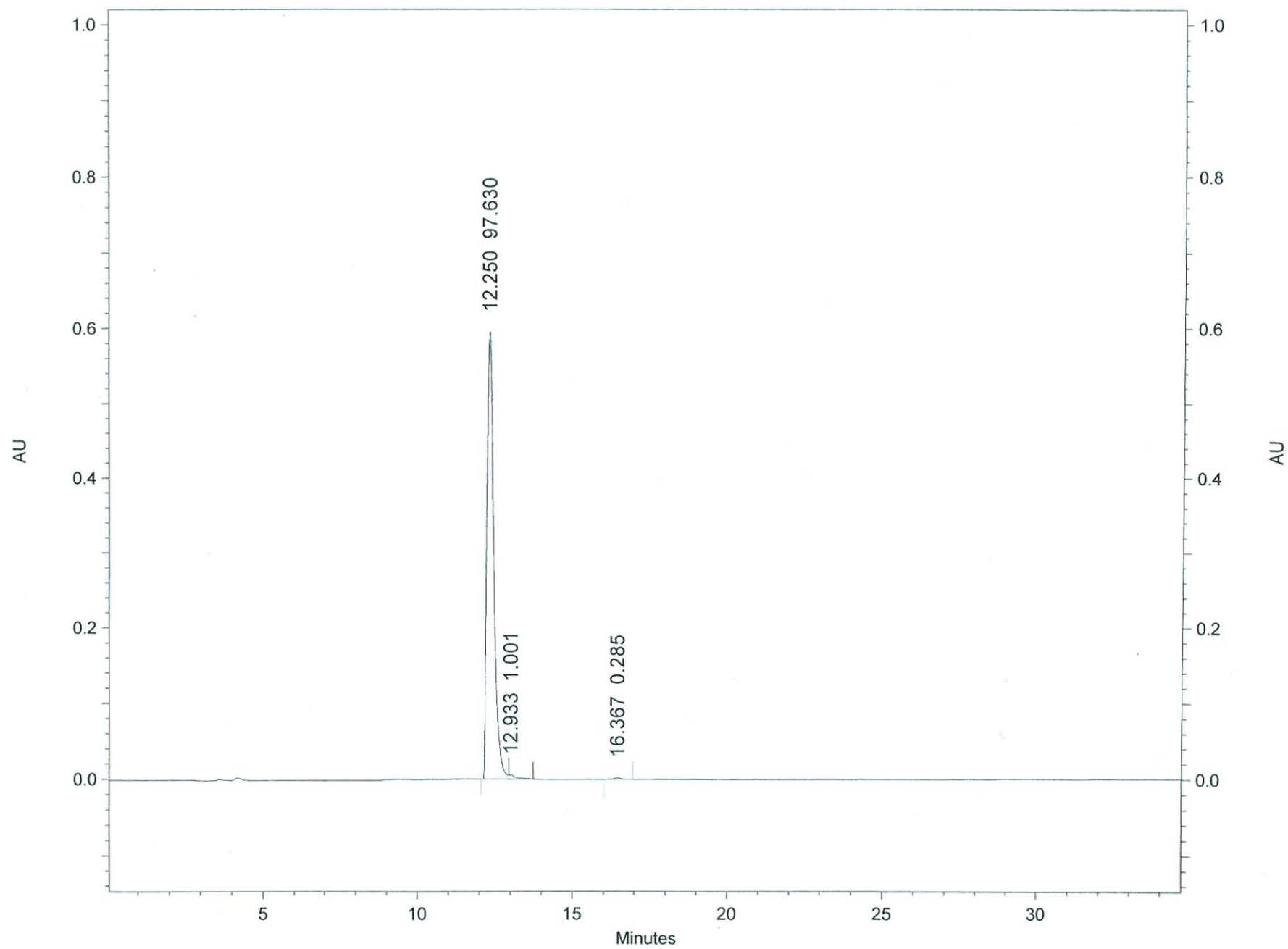

Supplement: Figure S1 — R16 HPLC chromatogram. (PDF) [file pone.0017883.s001.pdf]

# R16b HPLC Chromatogram

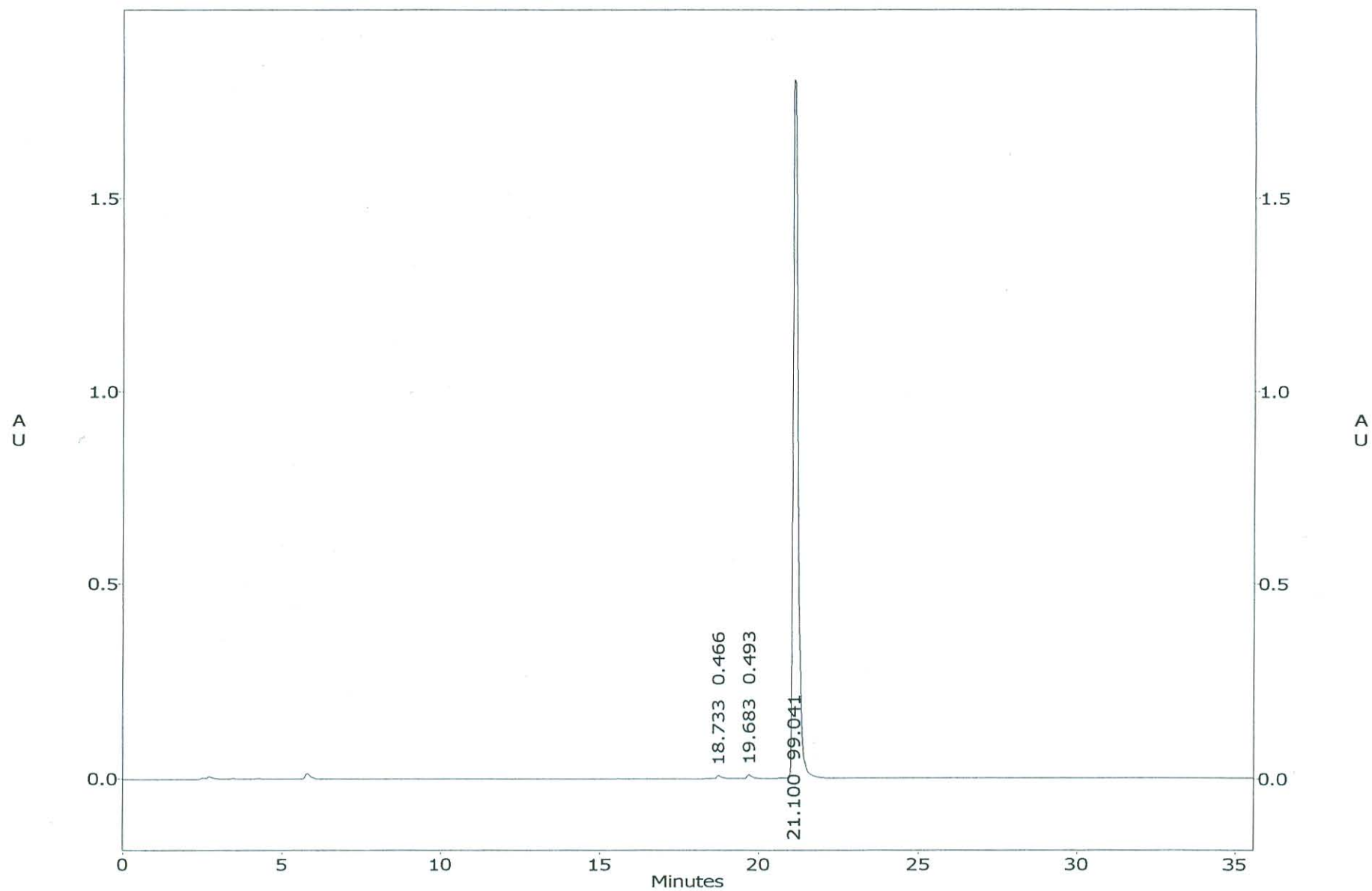

Supplement: Figure S2 — R16b HPLC chromatogram. (PDF) [file pone.0017883.s002.pdf]
